# 

**Published:** 1841-04

**Authors:** 


### WESTERN JOURNAL

OF

### MEDICINE AND SURGERY.

### COLLABORATORS.

Edward H. Barton, M. D. *Professor of the Theory and Practice of Medicine, in the Medical College of Louisiana.*

William J. Barbee, M. D. *Cincinnati, Ohio.*

George W. Bayless, M. D. *Demonstrator of Anatomy in the Louisville Medical Institute.*

A. H. Buchanan, M. D. *Columbia, Tennessee.*

Charles Caldwell, M. D. *Professor of the Institutes of Medicine and Medical Jurisprudence in the Louisville Medical Institute.*

Samuel A. Cartwright, M. D. *Mississippi.*

A. Clapp, M. D. *New Albany, Indiana.*

Jedediah Cobb, M. D. *Professor of Anatomy in the Louisville Medical Institute.*

John Esten Cooke, M. D. *Professor of the Theory and Practice of Medicine in the Louisville Medical Institute.*

Samuel D. Gross, M. D. *Professor of Surgery in the Louisville Medical Institute.*

John Hardin, M. D. *Munfordsville, Ky.*

John P. Harrison, M. D. *late Professor of Materia Medica, in the Cincinnati College.*

John F. Henry, M. D. *Bloomington Illinois*

Samuel P. Hildreth, M. D. *Marietta, Ohio.*

Samuel Hogg, M. D. *Nashville, Tennessee.*

M. Z. Kreider, M. D., *Lancaster, Ohio.*

Moses L. Linton, M. D. *Springfield, Kentucky.*

Henry Miller, M. D. *Professor of Obstetrics and the Diseases of Women and Children in the Louisville Medical Institute.*

John W. Monette, M. D. *Washington, Mississsippi.*

Samuel B. Richardson, M. D., *Lecturer on Anatomy and Surgery, &c, Louisville, Ky.*

John L. Riddell, M. D. *Professor of Chemistry in the Medical College of Louisiana.*

Landon C. Rives, M. D. *late Professor of Obstetrics in the Medical Department of the Cincinnati College.*

Charles W. Short, M. D. *Professor of Materia Medica in the Louisville Medical Institute.*

G. Troost, M. D. *Professor of Chemistry and Mineralogy in the University of Nashville.*

Amasa L. Trowbridge, M. D. *Professor of Surgery, Willoughby University, Ohio.*

John A. Warder, M. D. *Cincinnati, Ohio.*

William Wood, M. D. *Cincinnati, Ohio*

### RECEIPTS FOR THE MEDICAL JOURNAL,

*For the month ending April* 1, 1841.

| | |
|---|---|
| Dr. Johnson, McConnelsville, Ohio, | $ 3 00 |
| E. C. Lathrop, Delta, Ohio, | 5 00 |
| E. P. Rucker, Murfreesboro, Tenn., | 5 00 |
| T. A. Hereford, Huntsville, Ala., | 5 00 |
| Watkins & Wilson, Brownsville, Tenn., | 5 00 |
| E. D. Pickett, Rodney, Miss., | 5 00 |
| C. A. Trimble, Chillicothe, Ohio, | 10 00 |
| B. Burnwell, Buffalo, N. Y., | 5 00 |
| D. McCall, Rome, Tenn., | 5 00 |
| J. S. Helm, Tuscumbia, Ala., | 10 00 |
| A. Leslie, Petersburg, Ia., | 5 00 |
| S. Hitchcock, Princeton, Ohio, | 2 00 |
| Barlow & Logan, Palestine, Ill., | 10 00 |
| N. L, Porter, Pembroke, Ky , | 5 00 |
| Wm. Walters, Belmont, Ohio, | 5 00 |
| A. B. Palmer, Harrisonville, Mo., | 5 00 |
| J. M. Morris, Somerset, Ohio, | 5 00 |
| L. Edwards, New Marion, Ia., | 3 00 |
| A. Kimball, Fort Henderson, Ala., | 10 00 |
| J. B. Berry, Hartford, Ky., | 5 00 |
| M. S. Reed, Hagerstown; Ia., | 5 00 |
| T. J. Montgomery, Maxville, Ky., | 5 00 |
| P. Wallace, Massilon, Ohio, | 5 00 |
| A. H. Smith, Sumpterville, Ala., | 10 00 |
| G. O. Pond, Columbus, Ill., | 5 00 |
| H. Alexander, Palestine, Ill., | 8 75 |
| S. Haynes, Owensboro, Ky., | 5 00 |
| Porter & Whitten, Rockford, Ia., | 5 00 |
| T. McGarraugh, Frankfort Ohio, | 5 00 |
| J. Lyons, Vienna, Ala., | 10 00 |
| W. B. McGuire, Waynesville, Ohio, | 5 00 |
| Harding, Manchester, Ia., | 5 00 |
| J. H. Harris, Clarksville, Tenn., | 5 00 |
| J. F. Summers, Washington, Ia., | 5 00 |
| Blackstone, Athens, Ohio, | 3 00 |
| C. Hansford, Knox, C. H., Ill., | 5 00 |
| J. B. Hinson, Trenton, Tenn., | 5 00 |
| W. A. Booth, Somerville, Tenn., | 1 50 |
| Higgason, " " | 7 50 |
| J. L. Williams, Camden, " | 5 00 |
| H. M. Brown, Newtown, Ohio, | 10 00 |
| J. Smick, Daswin, Ill , | 10 00 |
| Mrs M. McCornell Manchac, La., | 5 00 |
| Dr. A. Adams, N. Middletown, Ky., | 5 00 |
| J. Wilcox, Batesville, Ohio, | 3 00 |
| Underwood & Cook, Pendleton, Ia., | 5 00 |
| J. S. Sparks, Scipio P. O., Ohio, | 5 00 |
| J. C. Helm, Granville, Ia., | 5 00 |
| Miller, Hitesville, Ill., | 5 00 |
| D. R. Allison, Woodland Cottage, Ill., | 5 00 |
| S. Haynes, Owensboro, Ky., | 5 00 |
| J N. Dorsey, " " | 10 00 |
| S. Barber, Milroy, Ia., | 5 00 |
| B. F. Richardson; Pine Bluff, Ark., | 10 00 |
| Dr. A B. Price, Centreville Ohio, | 6 00 |
| R. S. Steele, Paris, Ill., | 5 00 |
| B F. Summers, Big Spring, Ky., | 5 00 |
| Kirby, Bellefonte, Ala., | 5 00 |
| Dryden, Hopkinsville, Ky., | 12 50 |
| P. Herod, Pleasant Shade, Tenn., | 5 00 |

To SUBSCRIBERS. The numerous receipts of the month still operate to prevent us from publishing a list of delinquents; and, as we remarked, in the March number, we shall not do so as long as the list of receipts continues respectable. There are still a great many delinquents, and it is a fact that up to this day, the outlay made on the Journal has not been refunded. We are sorry to say that the proportional number of delinquents in Kentucky is much larger than in any other State.

☞ Remit by mail, at our risk, under the frank of the Postmaster; who, under the regulations of the P. O. Department, will in all cases frank letters containing subscription money. See that the receipts of April do not fall short of those of March.

---

☞ No subscription to commence but with the commencement of a volume. No subscription can be discontinued until the end of the year, and none, after one number of a given volume has been mailed, until the end of the volume.

---

*To the Subscribers of the late* WESTERN JOURNANL OF THE MEDICAL AND PHYSICAL SCIENCES.

WE wish to inform such subscribers to the late "Western Journal of the Medical and Physical Sciences," Cincinnati, as paid for the 12th volume of that periodical, in advance, and received only one number, before it was discontinued, that by an arrangement with the publishers, they have been credited on our Journal for two dollars. One number of that work, amounted, it is true, only to seventy-five cents, but if its publishers had credited the subscribers, who remitted them three dollars, with that sum and accounted to us for two dollars and twenty-five cents, there could not have been any mode devised by which the remainder of our annual subscription, two dollars and seventy-five cents, could have been remitted. We are requested to say that should any of the old subscribers have an opportunity of applying to Dr. Drake or Dr. Rives, the publishing committee of that work, they will refund the twenty-five cents.

PRENTICE & WEISSINGER.

---

The WESTERN JOURNAL OF MEDICINE and SURGERY is published monthly by the undersigned, at the corner of Main and Fifth streets, Louisville, at $5 per annum, payable in advance. Each number contains from 80 to 84 pages making two volumes in the year of about 500 pages.

Contributions to its pages by the Physicians of the Valley of the Mississippi, addressed to the Editors, are respectfully solicited.

Letters on business, to be addressed, postage paid, to the publishers.

☞ Postmasters, by the regulations of the Postoffice Department, will frank letters containing subscription money, and all remittances so franked are at the risk of the publishers.

*July* 25, 1840. PRENTICE & WEISSINGER

